# Supplementary material for: Depressive episode and treatment outcomes in elderly individuals with tuberculosis: A prospective cohort study in Korea
Source: PLoS One. 2025 Nov 6;20(11):e0335897. doi: 10.1371/journal.pone.0335897 (PMC12591446; doi:10.1371/journal.pone.0335897)
Supplement: S1 Table — (DOCX) [file pone.0335897.s001.docx]

**Supplemental table 1.** Multivariable predictors of depressive episodes (PHQ-9 ≥10) after excluding participants with prior depression

|  | Adjusted OR (95% CI) | P value |
| --- | --- | --- |
| Sex |  |  |
| Male | Reference | 0.554 |
| Female | 1.190 (0.668 – 2.121) |  |
| Age, years |  |  |
| ≤ 74 | Reference |  |
| 75 – 84 | 0.883 (0.477 – 1.634) | 0.691 |
| ≥ 85 | 0.821 (0.313 – 2.151) | 0.688 |
| Unemployment | 10.069 (1.311 – 70.303) | **0.026** |
| CCI score |  |  |
| 0 | Reference |  |
| 1 – 2 | 2.720 (1.167 – 6.336) | **0.020*** |
| ≥ 3 | 3.246 (1.182 – 8.916) | **0.022*** |
| Cough or sputum | 2.008 (1.083 – 3.721) | **0.027*** |
| Alarming symptoms | 1.747 (0.982 – 3.082) | 0.058 |
| Constitutional symptoms | 2.2356 (1.331 – 4.168) | **0.003*** |
