# Supplementary material for: Depressive episode and treatment outcomes in elderly individuals with tuberculosis: A prospective cohort study in Korea
Source: PLoS One. 2025 Nov 6;20(11):e0335897. doi: 10.1371/journal.pone.0335897 (PMC12591446; doi:10.1371/journal.pone.0335897)
Supplement: S2 Table — (DOCX) [file pone.0335897.s002.docx]

**Supplemental table 2.** Baseline characteristics of participants stratified by functional status

| Variables | | Functional impairment | | | | Total | | P value |
| --- | --- | --- | --- | --- | --- | --- | --- | --- |
|  |  | No | | Yes | |  |  |  |
|  |  | (n = 218) | | (n = 143) | | (n = 361) | |  |
|  |  | n | % | n | % | n | % |  |
| Female | Yes | 90 | 41.3 | 64 | 44.8 | 154 | 42.7 | 0.514 |
| Age, years | ≤ 74 | 103 | 47.2 | 63 | 44.1 | 166 | 46.0 | 0.818 |
|  | 75 – 84 | 92 | 42.2 | 65 | 45.5 | 157 | 43.5 |  |
|  | ≥ 85 | 23 | 10.6 | 15 | 10.5 | 38 | 10.5 |  |
| Ever smoker | No | 116 | 53.2 | 87 | 60.8 | 203 | 56.2 | 0.182 |
|  | Yes | 100 | 45.9 | 56 | 39.2 | 156 | 43.2 |  |
|  | missing | 2 | 0.9 | 0 | 0.0 | 2 | 0.6 |  |
| Living alone | No | 161 | 73.9 | 105 | 73.4 | 266 | 73.7 | 0.985 |
|  | Yes | 57 | 26.1 | 37 | 25.9 | 94 | 26.0 |  |
|  | missing | 0 | 0.0 | 1 | 0.7 | 1 | 0.3 |  |
| Unemployment | No | 29 | 13.3 | 17 | 11.9 | 46 | 12.7 | 0.676 |
|  | Yes | 186 | 85.3 | 125 | 87.4 | 311 | 86.1 |  |
|  | missing | 3 | 1.4 | 1 | 0.7 | 4 | 1.1 |  |
| CCI score | 0 | 54 | 24.8 | 28 | 19.6 | 82 | 22.7 | 0.499 |
|  | 1 – 2 | 129 | 59.2 | 89 | 62.2 | 218 | 60.4 |  |
|  | ≥ 3 | 25 | 11.5 | 26 | 18.2 | 61 | 16.9 |  |
| Depression | Yes | 3 | 1.4 | 4 | 2.8 | 7 | 1.9 | 0.338 |
| Diabetes | Yes | 66 | 30.3 | 45 | 31.5 | 111 | 30.7 | 0.810 |
| Chronic lung disease | Yes | 16 | 7.3 | 14 | 9.8 | 30 | 8.3 | 0.409 |
| Prior TB treatment | Yes | 41 | 18.8 | 22 | 15.4 | 63 | 17.5 | 0.402 |
| Severe TB disease | Yes | 73 | 33.5 | 53 | 37.1 | 126 | 34.9 | 0.486 |
| Rifampicin resistance | Yes | 7 | 3.2 | 4 | 2.8 | 11 | 3.0 | 0.823 |
| Cough or sputum | Yes | 123 | 56.4 | 84 | 58.7 | 207 | 57.3 | 0.663 |
| Alarming symptoms | Yes | 65 | 29.8 | 46 | 32.2 | 111 | 30.7 | 0.636 |
| Constitutional symptoms | Yes | 59 | 27.1 | 61 | 42.7 | 120 | 33.2 | 0.002 |
