# Supplementary material for: Depressive episode and treatment outcomes in elderly individuals with tuberculosis: A prospective cohort study in Korea
Source: PLoS One. 2025 Nov 6;20(11):e0335897. doi: 10.1371/journal.pone.0335897 (PMC12591446; doi:10.1371/journal.pone.0335897)
Supplement: S3 Table — (DOCX) [file pone.0335897.s003.docx]

**Supplemental table 3.** Multivariable logistic regression analysis to assess factors associated with functional impairment

| Variables | aOR | Lower 95% CI | Upper 95% CI | P value |
| --- | --- | --- | --- | --- |
| Female | 0.916 | 0.517 | 1.623 | 0.764 |
| Age, years |  |  |  |  |
| ≤ 74 | Reference |  |  |  |
| 75 – 84 | 1.048 | 0.654 | 1.68 | 0.845 |
| ≥ 85 | 1.058 | 0.504 | 2.22 | 0.882 |
| Ever smoker | 0.704 | 0.394 | 1.258 | 0.235 |
| Constitutional symptoms | 2.090 | 1.330 | 3.283 | 0.001 |
