# Supplementary material for: Depressive episode and treatment outcomes in elderly individuals with tuberculosis: A prospective cohort study in Korea
Source: PLoS One. 2025 Nov 6;20(11):e0335897. doi: 10.1371/journal.pone.0335897 (PMC12591446; doi:10.1371/journal.pone.0335897)
Supplement: S4 Table — (DOCX) [file pone.0335897.s004.docx]

**Supplemental table 4.** Baseline characteristics of participants stratified by suicidal ideation

| Variables | | Suicidal ideation | | | | Total | | P value |
| --- | --- | --- | --- | --- | --- | --- | --- | --- |
|  |  | No | | Yes | |  |  |  |
|  |  | (n = 290) | | (n = 71) | | (n = 361) | |  |
|  |  | n | % | n | % | n | % |  |
| Female | Yes | 120 | 41.4 | 34 | 47.9 | 154 | 42.7 | 0.320 |
| Age, years | ≤ 74 | 134 | 46.2 | 32 | 45.1 | 166 | 46.0 | 0.949 |
|  | 75 – 84 | 125 | 43.1 | 32 | 45.1 | 157 | 43.5 |  |
|  | ≥ 85 | 31 | 10.7 | 7 | 9.9 | 38 | 10.5 |  |
| Ever smoker | No | 161 | 55.5 | 42 | 59.2 | 203 | 56.2 | 0.620 |
|  | Yes | 127 | 43.8 | 29 | 40.8 | 156 | 43.2 |  |
|  | missing | 2 | 0.7 | 0 | 0.0 | 2 | 0.6 |  |
| Living alone | No | 217 | 74.8 | 49 | 69.0 | 266 | 73.7 | 0.297 |
|  | Yes | 72 | 24.8 | 22 | 31.0 | 94 | 26.0 |  |
|  | missing | 1 | 0.3 | 0 | 0.0 | 1 | 0.3 |  |
| Unemployment | No | 35 | 12.1 | 11 | 15.5 | 46 | 12.7 | 0.464 |
|  | Yes | 251 | 86.6 | 60 | 84.5 | 311 | 86.1 |  |
|  | missing | 4 | 1.4 | 0 | 0.0 | 4 | 1.1 |  |
| CCI score | 0 | 71 | 24.5 | 11 | 15.5 | 82 | 22.7 | 0.155 |
|  | 1 – 2 | 174 | 60.0 | 44 | 62.0 | 218 | 60.4 |  |
|  | ≥ 3 | 45 | 15.5 | 16 | 22.5 | 61 | 16.9 |  |
| Depression | Yes | 4 | 1.4 | 3 | 4.2 | 7 | 1.9 | 0.119 |
| Diabetes | Yes | 85 | 29.3 | 26 | 36.6 | 111 | 30.7 | 0.232 |
| Chronic lung disease | Yes | 24 | 8.3 | 6 | 8.5 | 30 | 8.3 | 0.962 |
| Prior TB treatment | Yes | 48 | 16.6 | 15 | 21.1 | 63 | 17.5 | 0.363 |
| Severe TB disease | Yes | 99 | 34.1 | 27 | 38.0 | 126 | 34.9 | 0.538 |
| Rifampicin resistance | Yes | 10 | 3.4 | 1 | 1.4 | 11 | 3.0 | 0.370 |
| Cough or sputum | Yes | 159 | 54.8 | 48 | 67.6 | 207 | 57.3 | 0.051 |
| Alarming symptoms | Yes | 78 | 26.9 | 33 | 46.5 | 111 | 30.7 | 0.001 |
| Constitutional symptoms | Yes | 90 | 31.0 | 30 | 42.3 | 120 | 33.2 | 0.072 |
