# Supplementary material for: Depressive episode and treatment outcomes in elderly individuals with tuberculosis: A prospective cohort study in Korea
Source: PLoS One. 2025 Nov 6;20(11):e0335897. doi: 10.1371/journal.pone.0335897 (PMC12591446; doi:10.1371/journal.pone.0335897)
Supplement: S5 Table — (DOCX) [file pone.0335897.s005.docx]

**Supplemental table 5.** Multivariable logistic regression analysis to assess factors associated with suicidal ideation

| Variables | aOR | Lower 95% CI | Upper 95% CI | P value |
| --- | --- | --- | --- | --- |
| Female | 1.277 | 0.733 | 2.226 | 0.389 |
| Age, years |  |  |  |  |
| ≤ 74 | Reference |  |  | 0.97 |
| 75 – 84 | 0.933 | 0.516 | 1.687 | 0.818 |
| ≥ 85 | 0.927 | 0.361 | 2.383 | 0.875 |
| CCI score |  |  |  |  |
| 0 | Reference |  |  | 0.208 |
| 1 – 2 | 1.631 | 0.774 | 3.438 | 0.198 |
| ≥ 3 | 2.256 | 0.914 | 5.564 | 0.077 |
| Depression | 4.411 | 0.894 | 21.766 | 0.068 |
| Cough or sputum | 1.669 | 0.933 | 2.986 | 0.085 |
| Alarming symptoms | 2.239 | 1.285 | 3.903 | 0.004 |
| Constitutional symptoms | 1.536 | 0.881 | 2.678 | 0.130 |
