# Supplementary material for: Depressive episode and treatment outcomes in elderly individuals with tuberculosis: A prospective cohort study in Korea
Source: PLoS One. 2025 Nov 6;20(11):e0335897. doi: 10.1371/journal.pone.0335897 (PMC12591446; doi:10.1371/journal.pone.0335897)
Supplement: S6 Table — (DOCX) [file pone.0335897.s006.docx]

**Supplemental table 6.** Distribution of treatment outcomes by depressive episode (PHQ-9 ≥10) among 350 participants with rifampin-susceptible tuberculosis.

| Treatment outcome | Depressive episodes | | | | Total | |
| --- | --- | --- | --- | --- | --- | --- |
|  | No | | Yes | |  |  |
|  | (n = 282) | | (n = 68) | | (n = 350) | |
|  | n | % | n | % | n | % |
| Treatment success | 223 | 79.1 | 44 | 64.7 | 267 | 76.3 |
| Treatment failed | 4 | 1.4 | 2 | 2.9 | 6 | 1.7 |
| Lost to follow-up | 6 | 2.1 | 2 | 2.9 | 8 | 2.3 |
| Died | 15 | 5.3 | 6 | 8.8 | 21 | 6.0 |
| Not evaluated | 16 | 5.7 | 7 | 10.3 | 23 | 6.6 |
| Still-on-treatment | 18 | 6.4 | 7 | 10.3 | 25 | 7.1 |
