# Supplementary material for: Depressive episode and treatment outcomes in elderly individuals with tuberculosis: A prospective cohort study in Korea
Source: PLoS One. 2025 Nov 6;20(11):e0335897. doi: 10.1371/journal.pone.0335897 (PMC12591446; doi:10.1371/journal.pone.0335897)
Supplement: Attachment — Submitted filename: Reviewer_Report_PONE-D-25-44015_Updated.docx [file pone.0335897.s007.docx]

# ****Reviewer: Jing Liang, MD. PhD****

# ****September 6, 2025****

To: PLOS One **Academic Editor**

# ****Cover Note to the Academic Editor****

# **I agreed to review this manuscript because the study addresses an important and underexplored issue in aging populations. Many elderly individuals today live with long-term health burdens from poor medical access in their youth, and this paper highlights how depression worsens tuberculosis (TB) outcomes in this vulnerable group. The research is scientifically sound, ethically compliant, and clearly presented. My recommendation is acceptance after minor revision, mainly encouraging the authors to polish English slightly and present male–female comparisons more explicitly for clarity.**

# ****Overall Summary**:** This is a well-designed and scientifically sound cohort study showing that depressive episodes negatively affect treatment outcomes in elderly tuberculosis patients. The study is important for aging populations and should be accepted after minor revision.

# Reviewer Report

Manuscript Number: PONE-D-25-44015

Title: Depressive episode and treatment outcomes in elderly individuals with tuberculosis: a prospective cohort study in Korea

Journal: PLOS ONE

## 1. Originality and Appropriateness

The manuscript presents results of an original prospective multicenter cohort study conducted in Korea (2020–2022). The findings have not been previously published elsewhere, and the study clearly addresses an important public health problem by examining the relationship between depressive episodes and tuberculosis treatment outcomes in elderly patients. The topic fits the scope of PLOS ONE. This research is particularly timely and needed for aging populations. After World War II, many individuals suffered from poor health due to lack of medical care in their youth. Now, as they reach older age, these long-term health consequences exacerbate disease burden. Addressing mental health in this context is of high importance.

## 2. Methodological Rigor

Study design: Prospective multicenter cohort across 13 hospitals, representative of different regions in Korea.
Sample: 361 elderly participants (≥65 years) with active TB. Clear inclusion/exclusion process shown in the flowchart.
Assessment: Depression was measured with the validated Korean version of the PHQ-9, with a cutoff ≥10 defining depressive episode.
Analysis: Logistic regression was applied to identify risk factors and associations with treatment success. Adjusted models included important covariates (age, sex, comorbidities, TB severity).
Ethics: IRB approval obtained (C19ONDI0458, XC20ENDI0086). Written informed consent documented.

The methodology is adequate, technically sound, and well described. Analyses are appropriate and reproducible. However, one suggestion for improvement: sex should be analyzed and presented more fully, not only by using “female” as a reference category. Explicit comparisons between males and females would make interpretation clearer for readers.

## 3. Results

Prevalence: 19.1% of participants had a depressive episode.
Risk factors: Unemployment (aOR 5.07), higher Charlson comorbidity score, cough, and constitutional symptoms.
Functional impairment: Increased with depression severity.
Suicidal ideation: Reported by 19.7%; associated with alarming TB symptoms.
Treatment outcomes: Depressive episode significantly reduced treatment success (64.7% vs. 79.1%, p=0.012). Independent association confirmed (aOR 0.478, 95% CI 0.261–0.878).
The results are clearly presented with supportive tables and figures. The statistical evidence supports the conclusions.

## 4. Discussion and Conclusions

The discussion appropriately interprets findings:
- Depression is common among elderly TB patients and linked with poor adherence, functional decline, and suicidality.
- Highlights the importance of routine depression screening at TB diagnosis.
- Discusses public health relevance and implications for integrated care.
- Limitations are acknowledged: self-reported PHQ-9, limited generalizability beyond elderly cohort.
Conclusions are supported by data, not overstated, and aligned with the study’s scope. The authors should consider revising the reporting of sex differences to make male–female comparisons explicit rather than leaving readers to infer.

## 5. Writing and Presentation

The article is written in standard English, intelligible and well structured.
Abstract accurately reflects the study.
Tables and figures are appropriate and clear.
References are up to date and relevant.
Minor language polishing could further enhance readability, but the manuscript already meets PLOS ONE standards.

## 6. Ethics and Data Availability

Ethics approval and informed consent are properly documented.
Data availability is described: de-identified datasets are accessible upon request via KNIH with IRB approval and data use agreement. This is compliant with PLOS ONE data policy.
No competing interests declared.
Funding disclosure complete.

## 7. Recommendation

The manuscript is scientifically sound, ethically compliant, and meets PLOS ONE criteria.
I recommend acceptance after minor revision, specifically encouraging the authors to (1) polish English slightly, and (2) report sex differences explicitly (male vs. female) for clearer interpretation.

## Short Reviewer Comments to the Academic Editor

This is an original, well-designed prospective cohort study addressing depression and tuberculosis outcomes in elderly individuals. The study is scientifically sound, methodologically rigorous, and written in clear English. The findings are important for aging populations, particularly given that many elderly individuals now suffering from disease were deprived of adequate healthcare in their youth (e.g., post–World War II generations). The results highlight how depressive episodes negatively impact functional status, suicidal ideation, and tuberculosis treatment success, making the case for integrated mental health screening at TB diagnosis.
I recommend acceptance after minor revision. The only improvements I suggest are (1) slight language polishing, and (2) reporting sex differences more explicitly by comparing males and females directly, rather than only presenting “female” as a category. These refinements would improve clarity for readers without altering the core findings.

**Confidential to Editor**
This is a well-conducted and much-needed study for the aging population, especially as many elderly individuals today carry long-term disease burdens from poor healthcare access in their youth (e.g., post–WWII generations). The study is methodologically sound, ethically compliant, and scientifically solid. My only concerns are minor: the authors should polish the English slightly and present sex differences more explicitly (male vs. female), rather than only listing “female.” Overall, I recommend acceptance after minor revision.
