# Supplementary material for: Depressive episode and treatment outcomes in elderly individuals with tuberculosis: A prospective cohort study in Korea
Source: PLoS One. 2025 Nov 6;20(11):e0335897. doi: 10.1371/journal.pone.0335897 (PMC12591446; doi:10.1371/journal.pone.0335897)
Supplement: Attachment — Submitted filename: PLOS ONE Re-review 10152025.docx [file pone.0335897.s009.docx]

# **Reviewer: Jing Liang, MD. PhD**

# **Oct 15^th^, 2025**

**To: PLOS Editor team:**

**PLOS ONE Re-Review Comments**

Manuscript Number: **PONE-D-25-44015R1**

Title: **Depressive episode and treatment outcomes in elderly individuals with tuberculosis: a prospective cohort study in Korea.**

**General Evaluation**

I have carefully reviewed the revised manuscript and the authors’ detailed responses. The revision has fully addressed all prior reviewer and editor comments. The manuscript now meets the scientific, ethical, and editorial standards required by PLOS ONE. The study is methodologically sound, the presentation is clear, and the revisions improve transparency and rigor. I recommend acceptance of the manuscript.

**Editor’s Comments**

1. Clarification of suicidal ideation prevalence (19.7%)

→ Revised and clarified that this percentage refers to all 361 participants. Clear statements added to Abstract and Results sections.

2. Reference accuracy on treatment adherence

→ Reference #8 updated to Panati et al., Clin Epidemiol Glob Health (2023), directly supporting poor adherence in TB patients with depression.

3. WHO definition of “cure” and reporting treatment failure

→ The revised manuscript now explicitly follows WHO guidance, clarifies the rationale for using a 9-month cutoff for treatment success, and provides a detailed outcome table (Supplemental Table 6).

4. Exclusion of rifampicin-resistant TB patients

→ Justified based on clinical heterogeneity and small sample size (3.0%, n=11). Explanation added to both Methods and Results sections.

5. Prior depression and antidepressant therapy

→ Defined as clinician-diagnosed and under active treatment; discussion of bias and sensitivity analysis excluding 7 such participants were added. Results unchanged.

6. Consistency of patient counts

→ Clarified that PHQ-9 <10 group (n=292) includes both 0–4 and 5–9 subgroups; explicit breakdown provided.

7. Additional variables suggested (BMI, regimen, region, etc.)

→ BMI and regimen type were added to models; other variables (region, AE data, adjunct therapy) were unavailable and acknowledged as a limitation.

8. Marking significant p-values

→ All tables now include asterisks (*) for significant results for improved readability.

**All editorial concerns have been fully resolved**.

**Reviewer #1 Comments**

1. Male–female comparison clarity

→ Added explicit sex-based comparisons in text and tables, including male/female counts and adjusted odds ratio (female a OR = 1.168, 95% CI 0.685–2.076).

2. Language quality

→ English refined for clarity, formatting standardized, and terminology harmonized (e.g., rifampicin).

Reviewer #1’s suggestions have been thoroughly addressed.

**Reviewer #2 Comments**

All ten major comments and one minor comment have been carefully addressed:

1. Clarified suicidal ideation base (19.7%) → Corrected and clarified.

2. Updated references for poor adherence → Completed.

3. WHO cure definition and treatment outcome table → Fully revised and justified.

4.	Exclusion of RR-TB → Clear rationale added.

5.	Prior depression and antidepressant therapy → Defined, analyzed, and discussed with sensitivity analysis.

6.	Added BMI and other covariates → Included and explained, unchanged conclusions.

7.	Prior depression fewer episodes issue → Explained due to active treatment and small sample size.

8.	RR-TB in Table 1 explanation → Clarified as descriptive baselines for all 361 participants.

9.	Count discrepancy (292 vs 205) → Corrected with explicit PHQ-9 breakdown.

10.	Treatment success denominator (350) → Explained exclusion of 11 RR-TB participants.

11.	Mark significant p-values → All tables revised accordingly.

Each issue raised by Reviewer #2 is now completely resolved.

**Conclusion**

The authors have provided clear, data-based responses and thorough revisions. All prior editorial and reviewer concerns have been fully addressed. The manuscript now presents a well-organized, scientifically rigorous, and clinically meaningful study.

Recommendation: **Accept.**
